# Association between aldehyde exposure and kidney stones in adults

**DOI:** 10.3389/fpubh.2022.978338

**Published:** 2022-10-10

**Authors:** Yang Chen, Xudong Shen, Guoxiang Li, Shaoyu Yue, Chaozhao Liang, Zongyao Hao

**Affiliations:** ^1^Department of Urology, The First Affiliated Hospital of Anhui Medical University, Hefei, China; ^2^Institute of Urology, Anhui Medical University, Hefei, China; ^3^Anhui Province Key Laboratory of Genitourinary Diseases, Anhui Medical University, Hefei, China

**Keywords:** environmental pollution, aldehydes, kidney stones, isopentanaldehyde, benzaldehyde

## Abstract

Environmental pollution sources may play a key role in the pathogenesis of nephrolithiasis, although the link between environmental aldehyde exposure and the incidence of nephrolithiasis is unclear. The researchers in this study set out to see whether adult kidney stone formation was linked to environmental aldehydes. We examined data from 10,175 adult participants over the age of 20 who took part in the 2013–2014 National Health and Nutrition Examination Survey (NHANES), which was a cross-sectional research. A logistic regression model was employed in this work to examine the relationship between aldehyde exposure and kidney stones, machine learning was utilized to predict the connection of different parameters with the development of kidney stones, and a subgroup analysis was performed to identify sensitive groups. After controlling for all confounding variables, the results revealed that isopentanaldehyde, benzaldehyde, and hexanaldehyde were risk factors for kidney stone formation, with odds ratio (OR) of 2.47, 1.12, and 1.17, respectively, and 95 percent confidence intervals (95% CI) of 1.15–5.34, 1.02–1.22, and 1.00–1.36. Kidney stones may be a result of long-term exposure to aldehydes, which may cause them to form. Environmental pollution-related aldehyde exposure might give a novel notion and direction for future study into the process of kidney stone production, even if the cause is yet unknown.

## Introduction

Kidney stones are one of the most common diseases of the genitourinary system, endangering human health, seriously interfering with people's daily work and life, and imposing a significant economic burden on the health-care system, particularly in industrialized countries, as a result of lifestyle and diet. Because of a shift in lifestyle, the prevalence of urolithiasis has progressively grown over the last several decades (1). It's worth mentioning that kidney stone production rates vary greatly across nations and areas. According to current epidemiological research, the prevalence of kidney stones in the United States is more than 10% (2), whereas it is 9 percent in Europe (3) and 5.8 percent in China (4). Unfortunately, its prevalence is anticipated to rise further due to a variety of variables, including global warming, since stone disease is more likely to develop in warmer places (5, 6), and other environmental pollution-related factors also lead to kidney stones. Rising incidence, such as increased cadmium exposure (7), increased lead, mercury, and arsenic exposure (8), as well as ethnic origin, age, and gender, all impact the incidence of kidney stones (9). It is true that urinary calculi are benign lesions in the short term, but they may cause serious consequences, such as blockage of the urinary system and infection, which can lead to death. As a result, figuring out the causes of nephrolithiasis and how to prevent it is a top priority for public health issue.

Few studies have examined the role of external environmental elements in kidney stone development in recent years, despite there have been an increasing number of research on the mechanism of kidney stone development in recent years. As previously said, exposure to certain harmful compounds in the environment influences the occurrence of kidney stones, and these toxic substances present in every aspect of our everyday lives, making us impossible to avoid them. As a result, environmental contamination was proven to cause kidney stones in the everyday lives of individuals. The search for a probable explanation for the rise in cases is essential.

Aldehydes are common organic molecules with a CHO group to which organisms are regularly exposed, both exogenously and endogenously formed. Exogenous aldehydes may be obtained from a variety of sources, including the combustion of organic compounds such as gasoline, nicotine, food additives (10), and so on. Paints, radiators, synthetic carpets, and other sources of indoor aldehydes are the most common (11). Fire and agricultural combustion (12), as well as odor exposure at trash transfer facilities (13), are the primary producers of aldehydes in the air. In metropolitan areas, automobile exhaust gas directly emits aldehydes and hydrocarbons into the air, resulting in aldehydes in the air. Hydrocarbons, an important source of chemicals, are transformed into aldehydes by photochemical oxidation processes (14, 15). Furthermore, research have demonstrated that aldehyde exposure is widespread during laboratory dissection experiments (16). Endogenous aldehyde sources are generated by normal cellular metabolic processes such as lipid oxidation, glucose metabolism, histone demethylation, and so on. Endogenous aldehyde compounds such as 4-hydroxynonenal and malondialdehyde may be formed by lipid peroxidation of mitochondria and plasma membranes under oxidative stress conditions (17, 18). Aldehydes are highly reactive electrophilic compounds that are potentially carcinogenic and mutagenic (19–21), and aldehyde exposure can harm human health by causing allergic hypersensitivity diseases, liver disease, neurodegenerative disease (22), cardiovascular disease (23), and diabetes (24), but few studies have linked aldehyde exposure to kidney stones. As a consequence, this research investigates the possible link between aldehydes and kidney stone production based on the NHANES database in order to uncover preventative and therapy targets for kidney stone epidemic illness.

## Methods and materials

### Design of research

Data from the National Health and Nutrition Examination Survey (NHANES), a nationally representative cross-sectional survey of the general population conducted at non–profit organizations in order to collect nationally representative data on population health and nutritional status. Data from a survey other data included demographics, body measures, blood pressure, creatinine-urine, standard biochemistry profiles, and urological data. Data on aldehydes were only available for the 2013-2014 cycle. The NHANES website (www.cdc.gov/nchs/nhanes/) has further information on this data. The Institutional Review Board of the Ethics Review Board of the National Center for Health Statistics provided ethical permission for this research, which utilized previously gathered public data.

### Population research

In 2013-2014, a total of 10,175 participants took part in the NHANES survey. The following were among the exclusion criteria: 1. No one knew about kidney stones (*n* = 4,417); 2. At least one aldehyde or more was present (*n* = 4,983); a total of 775 individuals were included in the final research, including 68 with kidney stones.

### Evaluation of aldehyde exposure

Automated analytical methods combining solid-phase microextraction (SPME), gas chromatography (GC), and high-resolution mass spectrometry (HRMS) with selective ion mass detection and isotope dilution techniques were used to assess aldehyde levels in blood. Only six aldehydes were found in more than 75 percent of the subjects (23, 25). Therefore, this investigation included isopentanaldehyde, benzaldehyde, butyraldehyde, heptanaldehyde, hexanaldehyde, and propanaldehyde.

### Variables under investigation

Direct interviews and medical center examinations were used to obtain covariates that may influence the association between aldehyde concentrations and the risk of kidney stones, such as age, gender, race, education level, marital status, physical activity, water intake, household poverty-to-income ratio (PIR), body mass index (BMI), drinking and smoking status, history of diabetes, history of hypertension, and laboratory test results. Age and PIR in the household were handled as continuous factors. The following categorical variables were used: Gender (Female, Male), Race (Mexican American, White, Black, Other), Educational Level (High school or equivalent, College or above), Physical Activity (None, Moderate, Vigorous), Alcohol Consumption (No, Yes), Diabetes History (No, Yes, Cutoff), Hypertension History (No, Yes), and Marriage (no, yes). Body mass index (kg/m2) is computed by dividing one's body weight (kg) by one's height (m) squared.

### Statistical procedure

Categorical, dichotomous, or continuous variables are used to record data. The standard deviation of the mean is used to represent distributed continuous data; count proportions are used for dichotomous and categorical variables. The chi-square test (categorical variables), one-way ANOVA (normally distributed continuous variables), or Kruskal-Wallis' *H* test were used to identify differences in clinical features across groups (skewed distribution continuous variables).

To examine the risk of variables related with kidney stone development, we applied machine learning to predict the influence of each research variable on kidney stone formation. In this investigation, we developed three logistic regression models to evaluate independent associations: (1) unadjusted, (2) slightly adjusted, and (3) adjusted for all covariates. All the covariates were selected on the basis of their clinical importance, the estimated variables change of at least 10% of potential confounding effects. The probable relationship of each aldehyde and kidney stones was then shown using a smooth curve fit. We used hierarchical multiple logistic regression to uncover sensitive groups using subgroup analysis. In addition, during the model development phase, we constructed an XGBoost algorithm model to predict the relative importance of the selected variables. We implemented the XGBoost model to analyze the contribution (gain) of each variable to the prevalence of kidney stones (26, 27). R 3.5.3 (http://www.r-project.org/) and Empower Stats software (http://www.empowerstats.com) were used for statistical analysis, and a *p* value of 0.05 was deemed statistically significant.

## Result

### The comparison of baseline data

The main features of baseline data between study arms are shown in Table 1. A total of 775 people took part in the study, including 68 kidney stone sufferers. The non-nephrolithic and nephrolithic populations had mean ages of 48.37 17.03 and 54.75 15.65 years, respectively. In terms of racial distribution, white participants made up the lion's share of the NHANES population. Non-nephrolithic and nephrolithic subjects differed considerably in terms of age, gender, blood pressure, and serum creatinine levels. Three aldehydes, Benzaldehyde (ng/mL), Hexanaldehyde (ng/mL), and Isopentanaldehyde (ng/mL), were found to be considerably higher in stone sufferers.

**Table 1:** Baseline characteristics of participants in NHANES from 2013–2014.

**Characteristic**	**Stone formers (*n* = 68)**	**Non–stone formers (*n* = 707)**	***P*-value**
Age (years)	54.75 ± 15.65	48.37 ± 17.03	0.003
**Gender**			0.043
Male	40 (58.82%)	325 (45.97%)	
Female	28 (41.18%)	382 (54.03%)	
**Race**			0.007
Mexican American	16 (23.53%)	198 (28.01%)	
White	41 (60.29%)	286 (40.45%)	
Black	8 (11.76%)	115 (16.27%)	
Other	3 (4.41%)	108 (15.28%)	
**Education**			0.219
Less than high school	20 (29.41%)	150 (21.22%)	
High school or equivalent	16 (23.53%)	155 (21.92%)	
College or above	32 (47.06%)	402 (56.86%)	
**Alcohol**			0.813
Yes	49 (73.13%)	481 (74.46%)	
No	18 (26.87%)	165 (254%)	
**Marital status**			0.861
Married	42 (61.76%)	429 (60.68%)	
Unmarried	26 (38.24%)	278 (39.32%)	
**Smoked**			0.392
Yes	33 (48.53%)	305 (43.14%)	
No	35 (51.47%)	402 (56.86%)	
**Blood pressure**			<0.001
Yes	37 (54.41%)	242 (34.23%)	
No	31 (459%)	465 (65.77%)	
**Physical activity**			0.376
Never	47 (69.12%)	442 (62.52%)	
Moderate	13 (19.12%)	135 (19.09%)	
Vigorous	8 (11.76%)	130 (18.39%)	
**Diabetes**			0.094
Yes	14 (20.59%)	82 (11.60%)	
No	52 (76.47%)	607 (85.86%)	
Borderline	2 (2.94%)	18 (2.55%)	
Ratio of family income to poverty	2.29 ± 1.62	2.59 ± 1.65	0.16
BMI (kg/m^2^)	29.55 ± 8.03	29.19 ± 6.88	0.683
Serum creatinine (mg/dl)	0.98 ± 0.47	0.87 ± 0.26	0.002
Urine creatinine (mg/dl)	122.70 ± 77.55	108.59 ± 76.06	0.145
Total water (ml)	2185.99 ± 2136.38	2401.23 ± 2097.66	0.457
Benzaldehyde (ng/mL)	2.29 ± 4.01	1.63 ± 1.68	0.01
Butyraldehyde (ng/mL)	0.58 ± 0.28	0.59 ± 0.34	0.829
Heptanaldehyde (ng/mL)	0.56 ± 0.43	0.51 ± 0.15	0.053
Hexanaldehyde (ng/mL)	3.29 ± 7.46	2.32 ± 1.02	0.001
Isopentanaldehyde (ng/mL)	0.77 ± 0.70	0.60 ± 0.49	0.012
Propanaldehyde (ng/mL)	2.24 ± 1.11	2.06 ± 0.89	0.125

Statistically significant: p < 0.05; BMI, body mass index.

### The data analysis by machine learnling

**Figure 1:**
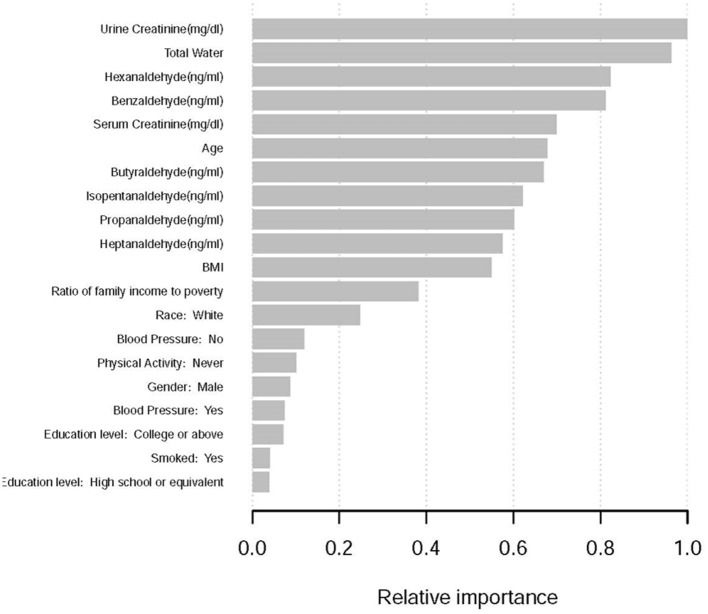
Importance of the variables in the machine learning model, scaled to a maximum of 1.

All variables were included in the machine learning model (28), and sorted according to the impact results, the results show that the top 10 factors influencing the formation of kidney stones include (Figure 1): urinary creatinine, water intake, Hexanaldehyde (ng/ml), Benzaldehyde (ng/ml) ml), Serum Creatinine (mg/dl), Age, Butyraldehyde (ng/ml), Isopentanaldehyde (ng/ml), Propanaldehyde (ng/ml), Heptanaldehyde (ng/ml).

### Multiple logistic regression analysis

**Figure 2:**
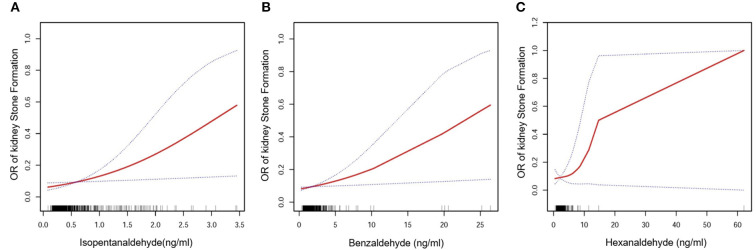
Isopentanaldehyde and total kidney stone formation dose-response relationship **(A)**. Benzaldehyde and total kidney stone formation dose-response relationship **(B)**. Hexanaldehyde and total kidney stone formation dose-response relationship **(C)**. The area between the upper and lower dashed lines is indicated as the 95% CI. The red line is connected by the magnitude of the aldehyde concentration into a continuous line. Adjustments were made for all covariates except for effect modifiers.

The major goal of this research was to determine the link between aldehyde exposure and the prevalence of kidney stone formation. A multiple logistic regression analysis was carried out. Three models were created using the Reporting of Strengthening Epidemiological Observational Studies (STROBE) standards (29): 1. Unadjusted model; 2. Model with minor adjustments; 3. Model with all variables adjusted. Table 2 shows that isopentanaldehyde/benzaldehyde and hexanaldehyde were strongly linked with the incidence of kidney stones, with OR, 95 percent CIs of 2.47 (1.15, 5.34) /1.12 (1.02, 1.22) /1.17 (1.00, 1.36), respectively. We also flattened the curve fitting in order to examine the association between the three aldehydes and kidney stones. The findings revealed that isopentanaldehyde and benzaldehyde had a linear connection (Figure 2), but hexanaldehyde had a non–linear relationship, with no evident threshold impact.

**Table 2:** Logistic regression analysis aldehydes exposure and kidney stones prevalence.

**Characteristic**	**Model1OR (95% CI)**	**Model2OR (95% CI)**	**Model3OR (95% CI)**
Isopentanaldehyde (ng/mL)	1.62 (1.10, 2.38)	1.71 (1.13, 2.60)	2.47 (1.15, 5.34)
Benzaldehyde (ng/mL)	1.10 (1.01, 1.19)	1.12 (1.03, 1.22)	1.12 (1.02, 1.22)
Butyraldehyde (ng/mL)	0.92 (0.42, 1.99)	0.94 (0.42, 2.08)	0.49 (0.13, 1.88)
Heptanaldehyde (ng/mL)	2.31 (0.87, 6.11)	3.89 (1.39, 10.92)	0.62 (0.08, 4.94)
Hexanaldehyde (ng/ml)	1.10 (0.97, 1.25)	1.13 (1.01, 1.27)	1.17 (1.00, 1.36)
Propanaldehyde (ng/mL)	1.21 (0.95, 1.55)	1.21 (0.95, 1.55)	0.83 (0.50, 1.38)

Model 1, no covariates were adjusted.

Model 2, Model 1+age, sex, race were adjusted.

Model 3, Model 2 + diabetes, blood pressure, education were adjusted. Not adjusted for the stratification variable itself.

### Subgroup analysis

We did a subgroup analysis of the three aldehydes based on the aforesaid findings (Table 3). The findings revealed that white people (OR = 5.08, 95% CI: 1.83–14.09) and those over the age of 50 (OR = 5.38, 95% CI: 1.39–20.78) were more likely to be white. The level of Isopentanaldehyde in the population was connected with the risk of kidney stones; female (OR = 1.15, 95% CI: 1.04–1.28), white (OR = 1.13, 95% CI: 1.04–1.28), and the level of Benzaldehyde in the population was associated with the risk of kidney stones. Only the female population in the Hexanaldehyde group was favorably linked with the incidence of nephrolithiasis (OR = 1.16, 95% CI: 1.03–1.56).

**Table 3:** Subgroup analysis between three aldehydes and kidney stones.

**Aldehydes**	**Subgroups**	**Model1OR (95% CI)**	**Model2OR (95% CI)**	**Model3OR (95% CI)**
Isopentanaldehyde (ng/mL)	**Stratified by sex**	
	Male	1.62 (0.98, 2.66)	1.98 (1.15, 3.40)	1.73 (0.59, 5.08)
	Female	1.52 (0.81, 2.85)	1.51 (0.77, 2.95)	3.69 (0.96, 14.22)
	**Stratified by race**	
	Mexican American	1.67 (0.38, 7.38)	1.11 (0.21, 5.79)	0.26 (0.01, 5.11)
	White	1.44 (0.92, 2.26)	1.63 (1.00, 2.66)	5.08 (1.83, 14.09)
	Black	2.15 (0.74, 6.24)	2.12 (0.72, 6.21)	0.00 (0.00, Inf)
	Other	0.29 (0.00, 21.72)	0.32 (0.00, 22.89)	0.00 (0.00, Inf)
	**Stratified by age**	
	AGE <50	1.75 (1.04, 2.94)	1.33 (0.76, 2.33)	5.38 (1.39, 20.78)
	AGE ≥50	1.87 (0.99, 3.50)	2.13 (1.08, 4.22)	2.53 (0.68, 9.47)
	Total	1.79 (1.20, 2.68)	1.70 (1.12, 2.56)	2.68 (1.23, 5.81)
Benzaldehyde(ng/ml)	**Stratified by sex**	
	Male	1.00 (0.75, 1.35)	1.06 (0.79, 1.42)	1.00 (0.72, 1.38)
	Female	1.13 (1.03, 1.22)	1.13 (1.03, 1.23)	1.15 (1.04, 1.28)
	**Stratified by race**	
	Mexican American	1.03 (0.82, 1.29)	1.07 (0.83, 1.36)	1.00 (0.74, 1.35)
	White	1.11 (1.01, 1.23)	1.14 (1.03, 1.27)	1.13 (1.01, 1.27)
	Black	1.34 (0.92, 1.94)	1.36 (0.91, 2.03)	1.00 (0.33, 3.00)
	Other	0.93 (0.36, 2.36)	0.92 (0.35, 2.37)	36.48 (0.00, Inf)
	**Stratified by age**	
	AGE <50	1.10 (0.99, 1.22)	1.09 (0.98, 1.22)	1.07 (0.95, 1.22)
	AGE ≥50	1.12 (0.98, 1.27)	1.20 (1.03, 1.40)	1.15 (0.97, 1.36)
Hexanaldehyde(ng/ml)	**Stratified by sex**	
	Male	0.98 (0.71, 1.37)	1.07 (0.78, 1.47)	1.16 (0.78, 1.71)
	Female	1.15 (0.92, 1.43)	1.15 (0.96, 1.37)	1.27 (1.03, 1.56)
	**Stratified by race**	
	Mexican American	0.77 (0.37, 1.59)	0.75 (0.33, 1.68)	1.40 (0.34, 5.82)
	White	1.16 (0.96, 1.41)	1.19 (0.98, 1.44)	1.21 (0.96, 1.54)
	Black	0.76 (0.36, 1.61)	0.73 (0.34, 1.55)	0.00 (0.00, Inf)
	Other	1.14 (0.89, 1.47)	1.15 (0.89, 1.47)	22.91 (0.00, Inf)
	**Stratified by age**	
	AGE <50	1.18 (0.92, 1.52)	1.16 (0.95, 1.41)	1.10 (0.87, 1.40)
	AGE ≥50	1.01 (0.76, 1.35)	1.05 (0.79, 1.39)	1.18 (0.84, 1.65)

The subgroup analysis was stratified by gender race and age.

## Discussion

Kidney stones, as we are all aware, are a common medical condition. People's eating habits and way of life are continually evolving in today's quickly emerging industrialized nations as a result of economic growth and rising living standards. Kidney stones are becoming more and more common. The prevalence of kidney stones is constantly rising. This not only causes a great deal of problems and annoyance in people's job and lives, but it also causes pain and devastation to health, as well as a significant strain on the social economy and the medical and health system. Although medical technology is advancing by leaps and bounds with the advancement of life science, clinical diagnosis and treatment methods for diseases are also changing with each passing day, and the pain and trauma suffered by clinical patients in the treatment of diseases is becoming smaller and smaller. However, medical science Progress has not slowed the rising occurrence of kidney stones from year to year. Many investigations on the mechanism of kidney stone formation have also been conducted, including the theory of renal calcium plaques, the theory of supersaturated crystallization, the theory of matrix, the theory of inhibitor shortage, and the theory of immunological damage (30). The hypothesis of supersaturation crystallization is the most researched mechanism, and its particular processes include supersaturation, crystal nucleation, aggregation, crystal development, deposition, and stone production (31). However, the specific mechanism of formation is still unknown, and these studies on the mechanism of formation of kidney stones are all at the molecular biology level, whereas there are few reports on the impact of the external environment that is closely related to human life on the formation of kidney stones, and it has not piqued people's interest.

Aldehydes are organic chemicals found in abundance in nature, and their origins are classified as exogenous and endogenous. Exogenous aldehydes enter the human body mostly *via* the respiratory system and the digestive tract (32), and are prevalent in people's everyday lives and workplaces. Endogenous aldehydes may be produced by lipid peroxidation, carbohydrate metabolism or ascorbic acid autoxidation, cytochrome P-450S, or metabolic activation mediated by myeloperoxidase (17, 18). Previous research has discovered that aldehyde exposure has negative effects on human health, including carcinogenicity, mutagenicity, cardiovascular disease, liver disease, embryotoxicity/teratogenicity, diabetes/hypertension, cerebral ischemia/toxicity in neurodegenerative diseases, and other aging-related diseases (19, 20). However, there have been no studies linking aldehyde exposure to kidney stones.

Serum aldehydes and kidney stones have never before been studied in a representative sample of the American population, according to our literature analysis. After controlling for all possible confounders, we discovered that Isopentanaldehyde/Benzaldehyde and Hexanaldehyde were positively linked with the incidence of kidney stones. Isopentanaldehyde/Benzaldehyde and Hexanaldehyde were shown to be linearly and non-linearly linked with the incidence of nephrolithiasis, respectively, in the findings.

Isopentanaldehyde has been linked to the onset of a variety of disorders in recent years, including obesity (25), cardiovascular disease (23), diabetes (24), and others. There is evidence that high-fat diets cause kidney injury in animals. Urinary oxalate and calcium levels were elevated as a result of crystal retention in the urothelium (33), and male Otsuka Long-Evans Tokushima fat (OLETF) rats were used to create an animal model of metabolic syndrome after drinking 1.0 percent ethylene glycol. The rats in the model group developed higher calcium oxalate crystal deposits after being exposed to water (34). Interestingly, it has been shown that the generation of glyoxal, a substrate for the synthesis of oxalic acid molecules, is increased during diabetic atherosclerosis. Furthermore, lipids include unsaturated fatty acids, and lipids are oxidized to create hydroperoxides, which are then converted to glyoxal (35). Oxalic acid levels that are too high may induce kidney injury and inflammation, both of which are required for the production of kidney stones. Furthermore, triglyceride production in the liver is linked to the de novo synthesis of purines, which are catabolized to generate uric acid, and metabolic syndrome is related with higher plasma uric acid levels (36). The elevated uric acid level promotes the production of uric acid crystals and the creation of uric acid stones. Although uric acid is often utilized as an antioxidant, it should be highlighted that xanthine oxidase (XO) produces reactive oxygen species during the synthesis of uric acid, which leads to the development of kidney stones (37). Furthermore, in patients with cardiovascular disease, there is a systemic imbalance between the essential fatty acid-3 and−6 pathways, which is thought to lead to increased levels of arachidonic acid phospholipids as well as hypercalciuria and hyperoxaluria, both of which are prerequisites for kidney stone formation. Obesity was shown to be positively linked with kidney stone development in the Taylor trial, with men with a BMI of 30 or higher having a 1.33 risk factor for kidney stones compared to men with a BMI of 21 to 22.9, while the risk factor for the same category of BMI in elderly and young women was 1.90 (38). And other studies have shown that a greater BMI has been linked to an increased risk of kidney stones (39), as have studies showing that those with diabetes are more likely to develop kidney stones (40). Finally, the data suggests that obesity, cardiovascular disease, and diabetes are risk factors for the development of kidney stones, and that isopentanaldehyde is linked to the development of these disorders. According to our findings, isopentanaldehyde is also a risk factor for the development of kidney stones. We suspect that it is the explanations might be multi-system and multi-faceted, and the precise process must be validated *via* additional *in vitro* and *in vivo* investigations.

Weng and colleagues used data from 1,795 participants in the National Health and Nutrition Examination Survey (NHANES) from 2013 to 2014 to conduct a multivariate logistic regression analysis of the relationship between aldehydes and diabetes. They discovered that benzaldehyde and hexanaldehyde may increase the risk of diabetes. Women's diabetes development is accelerated by their diabetes risk (24). Our findings reveal that benzaldehyde and hexanaldehyde are risk factors for kidney stone development, and that diabetes is also a risk factor. The underlying process of stone formation might be linked to the development of diabetes, and the particular molecular mechanism has to be confirmed.

To find out whether particular characteristics affect the likelihood of kidney stones, this research applied machine learning. Machine learning is widely used because of its advantages such as easy identification of trends and patterns, no human intervention, and continuous improvement. In this study we used XGboost machine learning to predict the relative importance of the effect of incorporated variables on the prevalence of kidney stones. Data may be ranked using machine learning depending on the significance of the information. For example, urine creatinine, daily water consumption, benzaldehyde and butyraldehyde concentrations as well as serum creatinine and age all play an important role in the kidney stone formation model. According to machine learning findings, the link between Butyraldehyde and the incidence of kidney stones is stronger than the correlation between Isopentanaldehyde and the occurrence of kidney stones. However, the results of our traditional logistic regression analysis indicate that butyraldehyde has no correlation with the occurrence of kidney stones. We hypothesize that this result is due to the small sample size used in the statistics, and to compare the performance of machine learning methods and traditional regression techniques. There have been several studies conducted on the advantages and disadvantages. However, the findings achieved so far have been rather disparate. Some studies have demonstrated that logistic regression may be as accurate or even more accurate than other machine learning techniques (28). Some research has indicated that machine learning approaches are more reliable than classical regression analysis, however. A distinct set of conclusions may be drawn when using the same strategy to other study subjects and datasets. A larger sample size might provide more accurate results, but this study's findings are also reassuring.

As a worldwide epidemic illness with a high prevalence, kidney stones have a significant influence on human health, but aldehyde exposure in environmental pollution and the formation of kidney stones have been overlooked. This research conclusion provides a new way of thinking and direction for the majority of researchers. An inadequate etiology for nephrolithiasis has been shown in this research, due to its cross-sectional design and the inability to establish a causal association between serum aldehydes and kidney stones. We must also take into account that our statistical findings can be influenced by the size of the sample and the sources of serum aldehydes. A rise in serum aldehydes can't be traced back to a specific source despite exposure to both internal and external sources of aldehydes, as shown by NHANES data.

## Conclusions

It's possible that aldehyde exposure is a contributing factor in kidney stones. Research on kidney stone formation's process may provide a fresh concept and direction if it focuses on aldehyde exposure caused by environmental pollutant pollution.

## Data availability statement

The datasets presented in this study can be found in online repositories. The names of the repository/repositories and accession number(s) can be found below: https://www.cdc.gov/nchs/nhanes/.

## Author contributions

YC: conceptualization, methodology, and software. XS: data curation and writing original draft. GL: visualization and investigation. SY: supervision and software. CL and ZH: writing—review and editing. All authors contributed to the article and approved the submitted version.

## Funding

This work was supported by the National Natural Science Foundation of China (82070724).

## Conflict of interest

The authors declare that the research was conducted in the absence of any commercial or financial relationships that could be construed as a potential conflict of interest.

## Publisher's note

All claims expressed in this article are solely those of the authors and do not necessarily represent those of their affiliated organizations, or those of the publisher, the editors and the reviewers. Any product that may be evaluated in this article, or claim that may be made by its manufacturer, is not guaranteed or endorsed by the publisher.
